# Evaluation of Odor and Physicochemical Properties in Sheep Placenta Processed With Different Drying Methods

**DOI:** 10.1002/fsn3.71641

**Published:** 2026-03-15

**Authors:** Jing Zhu, Yuqing Fan, Jiale Du, Xingxing Liu, Guisheng Yi, Hongmin Yu, Jingrong Fu, Jinghong Fu, Lingyun Zhong, Ming Yang

**Affiliations:** ^1^ Research Center for Traditional Chinese Medicine Resources and Ethnic Minority Medicine Jiangxi University of Chinese Medicine Nanchang China; ^2^ School of Pharmacy Jiangxi University of Chinese Medicine Nanchang China; ^3^ Jiangxi Tianyuan Pharmaceutical Co., Ltd. Yichun China; ^4^ National Key Laboratory of Classic Formula Modern Chinese Medicine Creation Nanchang China

**Keywords:** appearance, drying methods, odor compounds, physicochemical properties, sheep placenta

## Abstract

Sheep placenta is a functional food with proven pharmacological benefits, but its quality is often compromised by conventional drying methods. This study aimed to identify the optimal drying method for preserving the quality of sheep placenta by evaluating the effects of hot air drying (HAD), baking (BK), and vacuum freeze drying (VFD) on its appearance, physicochemical properties, and odor. Physicochemical properties were assessed using colorimetry, texture profile analysis, low‐field nuclear magnetic resonance, and amino acid profiling. Volatile compound analysis was performed by headspace gas chromatography–mass spectrometry coupled with an ultrafast gas chromatography electronic nose. Results showed that vacuum freeze dried samples maintained higher lightness, more intact microstructure, better retention of essential amino acids, and significantly lower fishy odor compared to samples treated with hot air drying or baking. Multimethod analysis (Mantel test, Pearson correlation, and TOPSIS) consistently identified vacuum freeze drying as the most effective process. These findings demonstrate that vacuum freeze drying is scientifically preferable for minimizing quality degradation in sheep placenta, providing valuable insights for its application in functional food processing.

## Graphical abstract

This study evaluated the effects of hot air drying, baking, and vacuum freeze drying on the quality of sheep placenta. Results showed that vacuum freeze drying better preserved the product's color, microstructure, and essential amino acid content while significantly reducing fishy odor. Comprehensive analysis consistently confirmed that vacuum freeze drying is the optimal drying method for minimizing quality loss in sheep placenta.

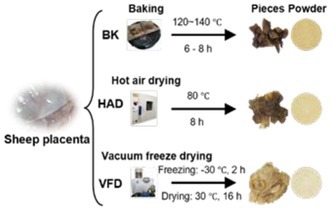

**Abbreviations**
A: alanine
a*: red‐green chromaticity
B*: blue value
b*: yellow‐blue chromaticity
BK: baking
BW: bound water
C: cysteine
*Ci*
: calculation of the closeness coefficient
D: aspartic acid
*D* ^+^_i_
: the distances of alternatives to positive ideal solution
*D* ^−^_i_
: the distances of alternatives to negative ideal solution
E: glutamate
E*: the total color difference
EAA: essential amino acids
F: phenylalanine
Fast GC E‐Nose: ultrafast gas chromatography electronic nose
FW: free water
G: glycine
G*: green value
H: histidine
H*: hue
HAA: hydrolyzed amino acids
HAD: hot air drying
HS‐GC‐MS: headspace gas chromatography–mass spectrometry
I: isoleucine
IW: immobilized water
K: lysine
L: leucine
L*: lightness value
LF‐NMR: low‐field nuclear magnetic resonance
M: methionine
NEAA: nonessential amino acids
P: proline
R: arginine
R*: red value
ROAV: relative odor activity value
S: serine
S*: saturation
SD: standard deviation
SEM: scanning electron microscopy
T: threonine
*T*_*2*_
: transverse relaxation time
TAA: total amino acid
V: valine
V*: value
VFD: vacuum freeze drying
Y: tyrosine

## 1 Introduction

Sheep placenta is the dried placenta of the bovid *Ovis aries* Linnaeus and is used as a functional food with medicinal value (Gansu Medical Products Administration 2021). Sheep are found across North America, Europe, Africa, and Asia (Yang et al. 2018). In China, they mainly grow in provinces such as Xinjiang, Sichuan, Inner Mongolia, and Shandong. Even though sheep placenta is rich in nutrients, it is usually thrown away as waste. The medicinal use of sheep placenta was first noted in Ben Jing Feng Yuan (Zhang 1996). Modern research has found that it is rich in active ingredients such as proteins, amino acids, enzymes, and trace elements, and has functions such as immunization regulation, anti‐oxidation, anti‐aging, and anti‐fatigue (Wang et al. 2018; Fan, Zhu, et al. 2024). In order to enhance the utilization value and broaden the application scope of sheep placenta and to mitigate unfavorable factors such as the inconvenience of transportation and the challenges associated with preserving fresh products, drying methods are employed to process sheep placenta. These methods reduce its moisture content, inhibit internal physiological changes, and thereby extend its shelf life (Bu et al. 2023; Li et al. 2023; Yang, Pu, et al. 2025).

During drying, many chemical reactions take place. These reactions can change how the product looks, its flavor, and its physical and chemical traits. This is also one of the important links that determine the flavor and quality of food (Fan, Guo, Zhu, et al. 2024; Zhong et al. 2024). In order to improve the quality of sheep placenta, drying is one of the most convenient and effective processing techniques. After drying, it is necessary to reduce the moisture content of the sample, maintain the stability of active ingredients, meet standard specifications, achieve uniform appearance color, and enhance its economic value. However, using high temperature to dry and process sheep placenta can easily denature and inactivate its active ingredients such as proteins and amino acids (Fu et al. 2024), affecting its medicinal effects. For example, in a comparison of peptide powders derived from porcine placenta hydrolysate prepared by vacuum drying, spray drying, and freeze drying, vacuum‐dried powders showed the lowest antioxidant activity and solubility. In contrast, freeze‐dried powders performed best across four antioxidant assays: DPPH, superoxide, hydroxyl radical scavenging, and lecithin liposome antioxidant activity (Tang et al. 2013). A comparison of the effects of vacuum freeze drying (VFD) and hot air drying (HAD) on placenta revealed that the vacuum freeze‐dried material, following water extraction, exhibited the highest efficacy in both preventing and treating anemia. This is attributed to the ability of VFD to preserve the activity of low‐molecular‐weight substances, an advantage not offered by HAD (Ren et al. 2022). The appearance of sheep placenta powder prepared by vacuum freeze drying, spray drying, and hot air drying was compared. The vacuum freeze‐dried product showed a dark red, flocculent morphology, while the hot air‐dried one appeared dark red with a dense, meat‐like texture (Wang 2019). Therefore, it is necessary to select an appropriate drying method and strictly control the drying process.

By reviewing the literature, the traditional drying methods for sheep placenta include sizzling drying, natural drying, roasting drying, and baking (BK). Modern methods mostly use hot air drying and vacuum freeze drying for processing. Meanwhile, HAD and VFD are also common methods for food and traditional Chinese medicine drying (Tang et al. 2025; Zhang et al. 2023; Zhao et al. 2025). However, both traditional and modern drying methods have their corresponding advantages and disadvantages. For example, BK is difficult to control the Charcoal Fire temperature, each drying sample requires a large amount of charcoal and the number of dried samples is limited, but the drying time is relatively short. The baking process requires operation by skilled personnel. Inexperienced handling can easily cause the samples to become scorched or even damaged. During the initial baking trials in this study, operational inexperience led to some wastage of sheep placenta samples. Ultimately, qualified samples were successfully prepared under the guidance of Professor Gong Qianfeng from Jiangxi University of Chinese Medicine; HAD is straightforward and easy to operate; however, the high temperatures involved can compromise surface integrity and lead to nutrient loss in the samples (Jin et al. 2024; Yuan et al. 2025; Zhang, Li, et al. 2025). Wang (2019) reported that no IgG content was detected in hot air‐dried sheep placenta powder. Similarly, in the present study, hot air drying was observed to compromise the structural integrity of sheep placenta; VFD can better preserve the thermosensitive components and flavor compounds in Substances, and produce products with good appearance, but it requires high energy consumption and a long drying time (Guo et al. 2024; Shen et al. 2023; Xiao et al. 2025). This study found that VFD can well preserve sheep placenta samples, with the color and appearance being relatively close to fresh sheep placenta, and IgG content can be detected (Wang 2019).

**FIGURE 1:**
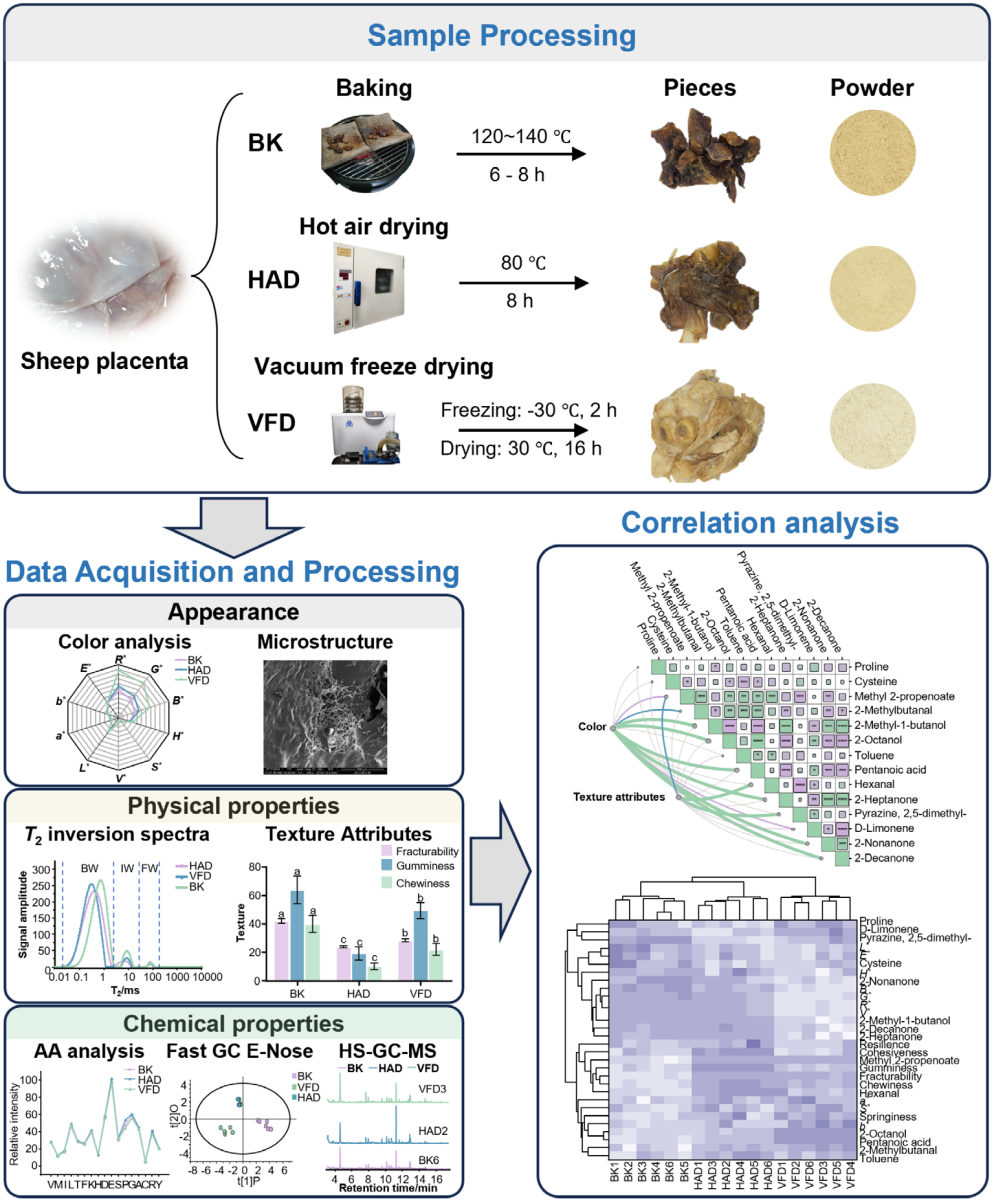
Flowchart of drying treatment and data analysis for different sheep placentas. a*, red‐green chromaticity (a*); A, alanine (A); AA analysis, amino acids analysis; B*, blue value; b*, yellow‐blue chromaticity; BK, baking; BW, bound water; C, cysteine; D, aspartic acid; E*, the total color difference; E, glutamate; F, phenylalanine; Fast GC E‐nose, ultrafast gas chromatography electronic nose; FW, free water; G*, green value; G, glycine; H*, hue; H, histidine; HAD, hot air drying; HS‐GC‐MS, headspace gas chromatography–mass spectrometry; I, isoleucine; IW, immobilized water; K, lysine; L*, lightness value; L, leucine; M, methionine; P, proline; R*, red value; R, arginine; S*, saturation; S, serine; T, threonine; T_2_, transverse relaxation time; V*, value; V, valine; VFD, vacuum freeze drying; Y, tyrosine.

Currently, to better explore the optimal drying method for sheep placenta, this study uses ultrafast gas chromatography electronic nose (Fast GC E‐Nose) and headspace gas chromatography–mass spectrometry (HS‐GC‐MS) to evaluate the flavor of sheep placenta, assesses the surface appearance and microstructure of sheep placenta based on colorimeter, camera color extraction, and scanning electron microscopy (SEM), and utilizes texture analyzer, low‐field nuclear magnetic resonance (LF‐NMR) technology, and amino acid analyzer to detect the physicochemical properties of sheep placenta. Finally, through the Mantel test, Pearson correlation analysis, and the TOPSIS comprehensive evaluation model, a multidimensional holographic quality evaluation system of “color‐component‐odor‐texture” for sheep placenta based on intelligent sensory recognition technology and chemometrics was constructed (Figure 1). The optimal drying method for sheep placenta was screened out, and a new comprehensive quality evaluation model for sheep placenta was constructed, providing a theoretical basis for the selection of the optimal drying method for sheep placenta and the improvement of its quality and industrial production.

## 2 Materials and Methods

### 2.1 Materials and Chemicals

Fresh sheep placentas were purchased from Inner Mongolia, Shaanxi and other provinces of China, transported to the laboratory by cold chain, and stored in a −20°C refrigerator for further use.

Amino acid mixed standard (TPE5585, purchased from Fuji Corporation in Japan), hydrochloric acid (20230911, purchased from Shanghai Lingfeng Chemical Reagent Co. Ltd.), Wahaha water.

### 2.2 Drying Process

Before drying, remove the sheep placenta from the refrigerator, thaw it, wash away the blood, remove the amnion and umbilical cord, and chop it into uniform pieces.

#### 2.2.1 HAD

Evenly spread the sheep placenta slices onto a tray and place them in an electric hot air oven (GZX‐9076MBE, Shanghai Boxun Industrial Co. Ltd. Medical Equipment Factory, Shanghai, China). Dry at 80°C for 8 h until the sheep placenta is completely dry.

#### 2.2.2 BK

Evenly spread the sheep placenta slices on the tiles, place the tiles on the grill and bake for 6–8 h. Control the temperature of the tiles at 120°C–160°C. When the temperature exceeds 160°C, the tiles need to be moved away from the grill. Turn over the sheep placenta every 15 min during the baking stage, keep the charcoal fire unextinguished and the temperature relatively constant without boiling over until the sheep placenta is completely dried.

#### 2.2.3 VFD

Evenly spread the sheep placenta slices in the material tray and place them in the cold trap of a vacuum freeze dryer (LGJ‐10E, Sihuan Furui Instrument Technology Development (Beijing) Co. Ltd., Beijing, China) for pre‐freezing for 2 h. After pre‐freezing, remove and dry them in the vacuum freeze dryer for 16 h at a drying temperature of 30°C (Fan, Zhu, Chen, et al. 2024).

### 2.3 Appearance

#### 2.3.1 Sample and Powder Colors

Color parameters of sheep placenta powder were measured using a color difference meter (NH300, Shenzhen Sanenchen Technology Co. Ltd., Shenzhen, China). The lightness value (L*), red‐green chromaticity (a*), and yellow‐blue chromaticity (b*) of the powder were recorded, and the total color difference (E*) was calculated using the formula (Zhang et al. 2023):

(1) $$E^{*}={\left(L^{*2}+a^{*2}+b^{*2}\right)}^{\frac{1}{2}}$$

Images of samples were captured using a digital camera (Nikon D750, Nikon Corporation, Japan). Red (R*), green (G*), and blue (B*) values were extracted from the images using ImageJ software, and the RGB values were normalized to the [0, 1] range. Hue (H*), saturation (S*), and value (V*) parameters were then converted from the normalized RGB values using the following formula:

(2) $$\begin{array}{c} \mathrm{Max}=\max\;\left(R^{*},G^{*},B^{*}\right),\mathrm{Min}=\min\;\left(R^{*},G^{*},B^{*}\right) \end{array}$$

(3) $$\begin{array}{c} \text{∆}=\mathrm{Max}-\mathrm{Min} \end{array}$$

(4) $$\begin{array}{c} V^{*}=\mathrm{Max} \end{array}$$

(5) $$\begin{array}{c} S^{*}=\left\{\begin{array}{cc} \frac{∆}{\mathrm{Max}}, & \text{if}\;\mathrm{Max}\neq \mathrm{Min}\\ 0, & \text{if}\;\mathrm{Max}=\mathrm{Min} \end{array}\right. \end{array}$$

(6) $$\begin{array}{c} H^{*}=\left\{\begin{array}{cc} \frac{\left(G^{*}-B^{*}\right)60}{∆}, & \mathrm{Max}=R^{*}\\ 120+\frac{\left(B^{*}-R^{*}\right)60}{∆}, & \mathrm{Max}=G^{*}\\ 240+\frac{\left(R^{*}-G^{*}\right)60}{∆}, & \mathrm{Max}=B^{*} \end{array}\right. \end{array}$$

#### 2.3.2 Microstructure of Sheep Placenta

The surface microstructure of the sheep placenta was determined using a scanning electron microscope (SEM, Quanta 250, FEI Company, USA). The prepared slices of sheep placenta using various drying methods were cut into 1 × 1 cm squares, adhered to the sample stage with conductive glue, and sputter‐coated with gold. The surface structure of the prepared slices was photographed at magnifications of 100×, 200×, and 500×.

### 2.4 Physical Properties

#### 2.4.1 Transverse Relaxation Time ( *T*_*2*_ ) Detection

Sheep placenta samples subjected to different drying treatments were placed into glass tubes with a 25 mm inner diameter. Low‐field nuclear magnetic resonance (LF‐NMR) analysis was performed using an NMI20‐025V‐1 analyzer (Suzhou NewMag Analysis Instrument Co. Ltd., Suzhou, China). The center frequency of the sample was obtained by an FID sequence. Six sets of data were collected for each group, and the peak ratio was analyzed after taking the average value. The main parameters of the *T*_*2*_ test included SF (MHz) = 20, RFD (ms) = 0.020, O_1_ (Hz) = 673139.11, RG1 (db) = 20.0, P1 (us) = 6.52, DRG1 = 3, TD = 60,006, PRG = 3, TW (ms) = 1000.00, NS = 16, P2 (us) = 12.48, TE (ms) = 0.150, and NECH = 2000. After the data were collected, the *T*_2_ of the samples was obtained by inversion processing using the instrument's software.

#### 2.4.2 Texture Attributes

The texture properties of sheep placenta samples treated with different drying methods were measured using a texture analyzer (TA.newplus, ISENSO, USA) equipped with a TA/1S probe. The test speed was set at 5 mm/s, with pre‐test and post‐test speeds of 2 mm/s and a compression deformation rate of 20%. Each drying method was measured six times, and the average value was taken for analysis.

### 2.5 Chemical Properties

#### 2.5.1 Hydrolyzed Amino Acid Detection

Take sheep placenta powder (300–600 mg), add it into a hydrolysis tube, add 10 mL of 6 M hydrochloric acid, blow nitrogen for 30 s and seal it. Place it in an oven at 110°C to hydrolyze for 22 h. After hydrolysis, complete the filtration and bring the volume to 100 mL in a volumetric flask. Take 2 mL of the volume‐fixed sample, lyophilize it to remove acid, add 2 mL of sodium citrate buffer to fully dissolve it, filter it through a 0.22 μm filter membrane, and analyze it on an amino acid analyzer (LA8080, Hitachi, Japan).

#### 2.5.2 Analysis of Flavor Compounds by Fast GC E‐Nose

The different dried placenta sample powders were detected using a fast gas phase electronic nose (Heracles NEO, Alpha MOS, France). 1.000 g of the sample powder that passed through a No. 3 sieve was precisely weighed and placed in a headspace vial, with 6 replicates per sample group.

Automatic sampling conditions were set as follows: incubation for 10 min at 60°C; stirring initiated for 1 s followed by a 20 s stirring pause; cleaning time of 90 s; syringe temperature maintained at 70°C; and filling speed set to 500 μL/s.

Detection conditions were set as follows: Chromatographic columns included an MXT‐1701 mid‐polar column and an MXT‐5 non‐polar column; injection volume was 3500 μL; injection flow rate was 125 μL/s; inlet temperature was 200°C; inlet pressure was 10 kPa; trap temperature was 40°C; initial column temperature was 50°C, programmed to increase at 1°C/s to 80°C, then at 3°C/s to 250°C with a 21‐s hold; acquisition time was 110 s; and data acquisition cycle was 0.01 s.

Retention times of sample chromatographic peaks were converted to retention indices, and qualitative analysis was performed by referencing the ArochemBase odor compound database (AlphaMos, Toulouse, France), yielding potential compound names and sensory descriptors.

#### 2.5.3 Analysis of Volatile Components by HS‐GC–MS

Headspace gas chromatography–mass spectrometry (HS‐GC‐MS) was carried out on a Mass‐hunter workstation (Agilent Technologies, USA) equipped with a 7697A headspace sampler, a 7890A gas chromatograph, and a 5975C mass spectrometer. Precisely weighed 1.0 g of sheep placenta powder was placed into 20 mL headspace vials, which were sealed with crimp caps. The heating oven temperature was set at 110°C, the quantitative loop temperature at 125°C, and the transfer line temperature at 150°C. Samples were equilibrated for 20 min, followed by a gas phase cycle time of 21 min (Yao et al. 2024).

The chromatographic column used was an HP‐5 quartz capillary column (0.25 mm × 30 m, 0.25 μm). A programmed temperature gradient was applied: an initial column temperature of 40°C held for 5 min, followed by heating at 8°C/min to 140°C. The inlet temperature was set at 280°C, with high‐purity helium as the carrier gas at a flow rate of 1.0 mL/min and a split ratio of 20:1.

The ion source was an electron impact ion source with an electron collision energy of 70 eV, an ion source temperature of 230°C, a quadrupole temperature of 150°C, a mass spectrometry interface temperature of 280°C, and the acquisition mode was full Scan mode.

Import the data obtained from HS‐GC‐MS detection into Data Analysis software, compare and retrieve it with the NIST 20.L mass spectrometry database, screen out the components with similarity ≥ 80%, and calculate the relative percentage content of each component using the area normalization method.

### 2.6 Statistical Analysis

All experimental results were replicated at least six times and presented as the mean ± standard deviation (SD). Data analysis and graphical representation were performed using Origin 2024 (Origin Lab, Northampton, MA, USA) and GraphPad Prism 10.1.2 (GraphPad Software LLC, San Diego, California, USA) Orthogonal partial least squares—discriminant analysis (OPLS‐ DA) was carried out using Simca 14.1 (Sartorius Stedim Data Analytics AB, Umeå, Sweden). Significant difference between samples was revealed by the analysis of variance followed by Duncan's multiple range test (*p* < 0.05) utilizing SPSS 18.0 software (SPSS Inc., Chicago, IL, USA).

## 3 Results

### 3.1 Appearance

#### 3.1.1 Color Analysis

**FIGURE 2:**
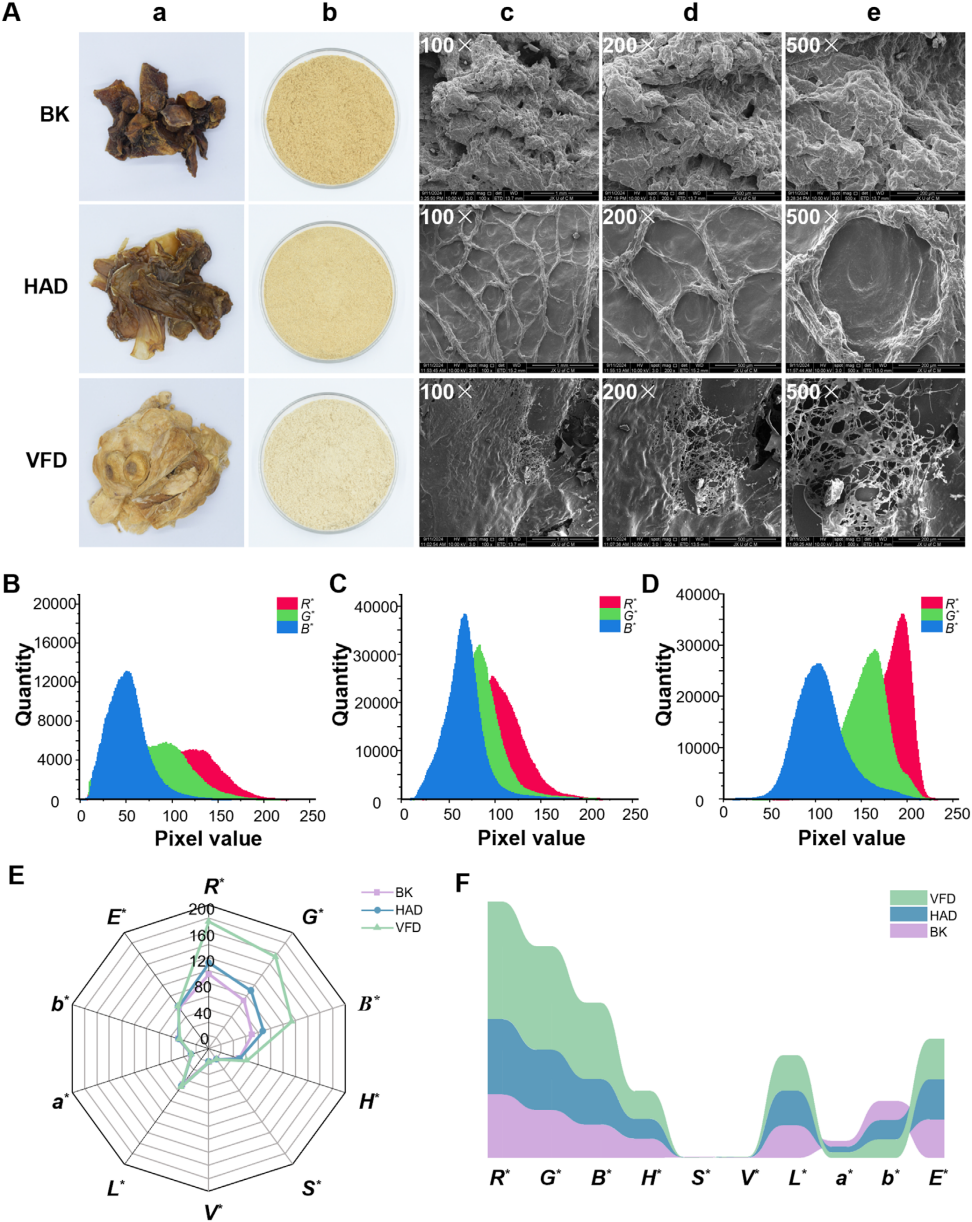
Different drying methods of sheep placenta slices, powders, scanning electron microscopes, and color influence diagrams. (A) Dried decoction pieces, powders and scanning electron microscope images. (a) Dried decoction pieces. (b) Powder. (c) 100× scanning electron microscope image with a scale of 1 mm. (d) 200× scanning electron microscope image with a scale of 500 μm. (e) 500× scanning electron microscope image with a scale of 200 μm. (B) The influence of different colors of BK samples. (C) The influence of different colors of HAD samples. (D) The influence of different colors of VFD samples. (E) Color radar chart. (F) Color change band diagram. a*, red‐green chromaticity; B*, blue value; b*, yellow‐blue chromaticity; BK, baking; E*, the total color difference; G*, green value; H*, hue; HAD, hot air drying; L*, lightness value; R*, red value; S*, saturation; V*, value; VFD, vacuum freeze drying.

Color is a critical sensory attribute for evaluating samples. Traditionally, color difference meters have been widely used to assess the color of powdered samples. However, this method often fails to fully characterize the extent of color changes in samples. To address this limitation, the present study utilized a camera to capture images of sheep placenta samples, extracted RGB values from sample surfaces, and converted them into HSV values, enabling a multidimensional evaluation of how different drying methods impact sample color. As shown in Figure 2A(ab), sheep placenta samples and powders processed by different drying methods exhibited distinct visual characteristics. BK samples displayed the darkest color with uneven distribution, while VFD samples presented the lightest color and relatively uniform appearance. The powder color was lighter than the color of the decoction pieces, because the internal color of the decoction pieces was lighter than the external color, and the color of the decoction pieces was more uniform after pulverization.

The effects of different drying methods on the color of sheep placenta were different, and the results are shown in Table 1. The L* of VFD samples was significantly higher than those of BK and HAD samples, with BK showing the lowest L*. This discrepancy can be attributed to the vacuum and low‐temperature conditions during VFD, which minimize oxidation reactions and enhance brightness. Additionally, the a* and b* of BK and HAD samples were higher than those of VFD samples, likely resulting from the combined effects of oxidation and Maillard reactions during thermal processing. Figure 2B–D illustrates the distribution of RGB pixel values on sample surfaces. Darker samples exhibited lower RGB values, and as sample lightness increased, corresponding RGB values rose proportionally (Li et al. 2018). Consequently, VFD samples showed significantly higher RGB values across all channels compared to the other two groups. Figure 2E,F depicts the color changes in samples and powders processed by different drying methods. VFD samples displayed the highest RGB values, along with the greatest hue and brightness, consistent with their high L*. In contrast, BK samples showed the highest saturation.

**TABLE 1:** The influence of different dry sheep placenta samples and powder colors, mean (*n* = 6) ± SD.

Drying methods	BK	HAD	VFD
L*	48.21 ± 2.79^b^	51.63 ± 1.89^a^	52.85 ± 1.88^a^
a*	8.92 ± 0.55^a^	8.28 ± 0.46^ab^	8.08 ± 0.68^b^
b*	29.34 ± 0.44^a^	28.74 ± 1.03^a^	27.06 ± 1.30^b^
E*	57.15 ± 2.29^b^	59.68 ± 0.84^ab^	59.93 ± 1.94^a^
R*	94.39 ± 3.67^c^	112.26 ± 13.31^b^	175.20 ± 8.74^a^
G*	71.33 ± 3.91^c^	89.72 ± 10.28^b^	154.36 ± 7.27^a^
B*	49.81 ± 5.57^c^	67.68 ± 6.05^b^	113.83 ± 7.35^a^
H*	28.72 ± 3.03^b^	29.41 ± 11.26^b^	41.08 ± 11.13^a^
S*	0.47 ± 0.06^a^	0.39 ± 0.08^ab^	0.35 ± 0.07^b^
V*	0.37 ± 0.01^c^	0.44 ± 0.05^b^	0.69 ± 0.04^a^

*Note:* Significant differences among samples were determined by analysis of variance and Duncan's multiple range test (*p* < 0.05), with different letters indicating statistically different values.

Abbreviations: a*, red‐green chromaticity; B*, blue value; b*, yellow‐blue chromaticity; BK, baking; E*, the total color difference; G*, green value; H*, hue; HAD, hot air drying; L*, lightness value; R*, red value; S*, saturation; SD, standard deviation; V*, value; VFD, vacuum freeze drying.

#### 3.1.2 Microstructure

Different drying methods have great differences on the changes of microstructure of sheep placenta processed slices. Figure 2A(cde) are scanning electron microscope images of sheep placenta under different magnifications with different drying methods. The drying process may lead to the formation of pressure differences within the sample structure, contributing to morphological changes (Wang et al. 2024). During BK process, sheep placenta samples are exposed to unstable high temperatures, leading to severe damage to their tissue structure. The structure in VFD and HAD samples is more compact, which may be attributed to the stable heating for drying process, resulting in more regular cell shrinkage and collapse (Zhang, Chen, Lan, et al. 2024). Since the VFD process involves continuous temperature increase, and the final drying temperature is the lowest among the three drying methods, the tissue structure of the sheep placenta after freeze‐drying is the most complete. Heating in a vacuum causes the water in the sample to sublimate from solid to gaseous state, thus leaving voids in the sample (Feng et al. 2022). A honeycomb pore structure can be observed on the sample surface, which makes it easier to dissolve active ingredients and improve bioavailability.

### 3.2 Physical Properties

#### 3.2.1 *T2* Inversion Spectra

Three distinct peaks in the *T*_2_ inversion spectrum distribution correspond to three water populations in sheep placenta samples, each representing a specific water state: *T*_21_ (0.1–1 ms) for bound water, *T*_22_ (1–10 ms) for immobilized water, and *T*_23_ (10–1000 ms) for free water (Tang et al. 2022). The peak integral areas reflect the relative proportions of these three water states. Larger peak areas signify higher water content in the corresponding state.

**FIGURE 3:**
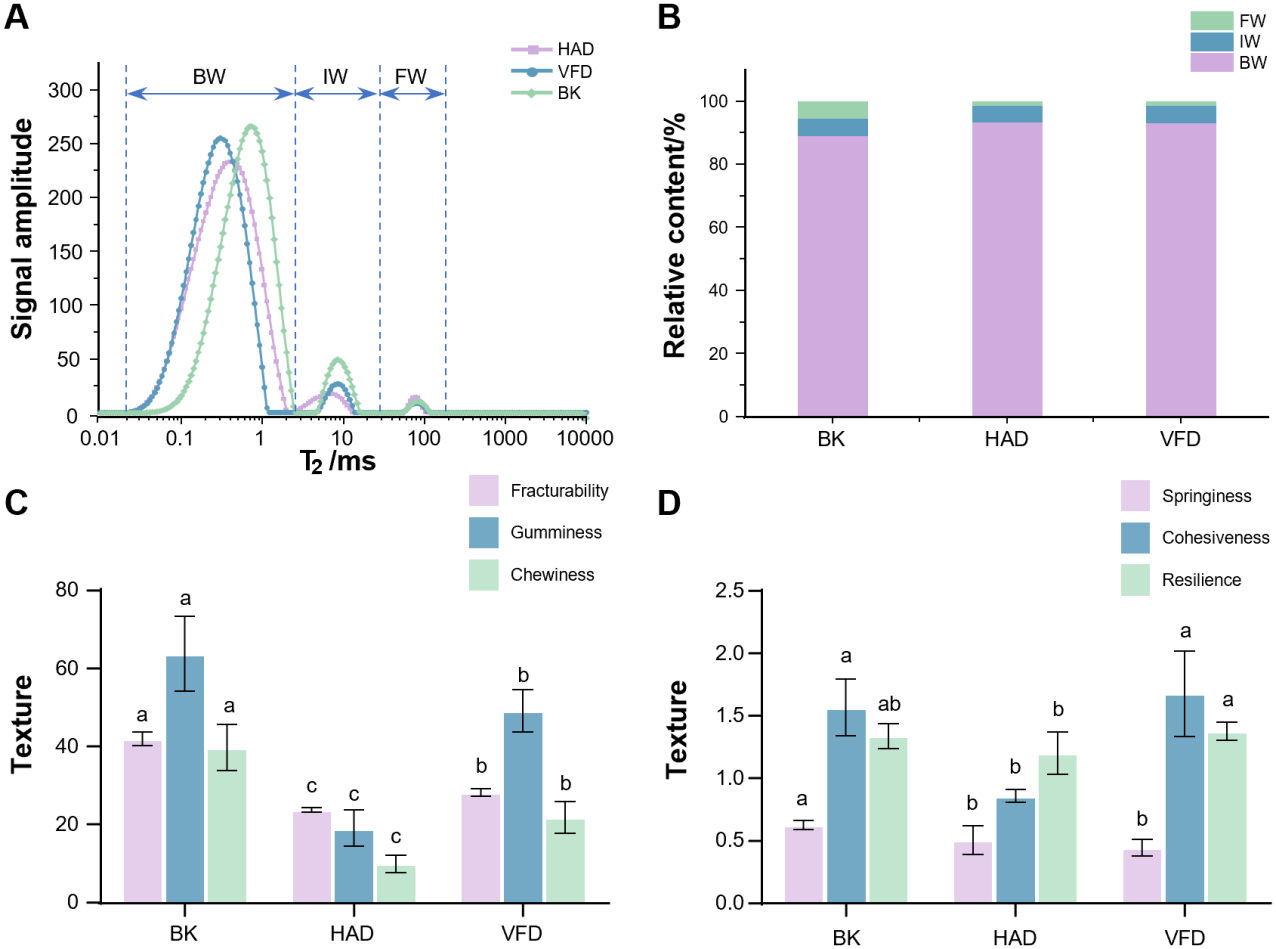
Low‐field nuclear magnetic resonance and texture property maps of placentas from different dried sheep. (A) *T*_2_ inversion spectra. (B) Peak area ratio. (C) The results for fracturability, gumminess, and chewiness. (D) The results for springiness, cohesiveness, and resilience. BK, baking; BW, bound water; HAD, hot air drying; FW, free water; IW, immobilized water; *T*_2_, transverse relaxation time; VFD, vacuum freeze drying. C and D uses mean (*n* = 6) ± SD. Significant differences among samples were determined by analysis of variance and Duncan's multiple range test (*p* < 0.05), with different letters indicating statistically different values.

The *T*_2_ value reflects the mobility of the water, smaller values of *T*_2_ indicate a more restricted mobility, whereas larger values of *T*_2_ indicate a greater degree of freedom of the water. Figure 3A shows the transverse relaxation time (*T*_2_) distribution of sheep placenta samples treated with different drying methods. As shown in Figure 3B, the BK samples had the highest total peak area, indicating the highest total water content, while the HAD samples had the lowest and the smallest total water content. The VFD samples showed intermediate water content, and the difference with the samples from HAD was not much. The three drying methods all had a higher percentage of bound water, indicating that sheep placenta was effectively dried after treatment by the three drying methods. The difference between the percentage of free water and not‐easy‐to‐flow water of BK was not big, although the BK sample was processed at a high temperature for a long time, but it was still difficult to remove the free water sufficiently. Compared with BK, HAD and VFD could remove the free water in the sample well, and the free water was all in a very low percentage after the drying was completed.

#### 3.2.2 Texture Attributes

The internal physicochemical properties of sheep placenta change after drying by different methods. The texture attributes of different dried sheep placenta slices were detected by texture analyzer, and the specific effects are shown in Figure 3. Among them, Figure 3C shows the results of fracturability, gumminess, and chewiness of different sheep placenta samples, and Figure 3D shows the results of springiness, cohesiveness, and resilience of different sheep placenta samples. Fracturability is defined as the maximum compressive force applied from the initial probe‐sample contact until sample fracture; a lower fracturability value indicates a crispier texture (Hu et al. 2024). HAD exhibited the lowest fracturability and greatest crispiness, attributed to high‐temperature heating during drying. This process induces rapid loss of surface free water and an increase in bound water, leading to the quick formation of a hardened surface layer on sheep placenta samples. The freeze‐drying process occurs under vacuum conditions, which prevents oxidative reactions and generates a porous structure in the sample, leading to intermediate fracturability in dried samples. By contrast, although BK process removes most free water, the residual free water proportion remains higher than that of the other two methods, resulting in the highest fracturability value and relatively lower crispiness compared to the other samples.

Chewiness is the work needed to chew solid food until it can be swallowed, and gumminess is the work needed to chew semisolid food. Both followed the same trend as fracturability (Wang et al. 2024). BK showed the least crispiness alongside the greatest chewiness and gumminess, while HAD was the crispiest with the lowest values for these attributes. Springiness, the ability of food to revert to its original shape after external force is removed, showed little difference among the three drying methods. Notably, BK samples exhibited slightly higher elasticity than VFD and HAD samples. Cohesiveness was significantly higher in BK and VFD compared to HAD. Resilience values of the three sample groups were comparable, possibly due to the sufficient moisture removal at the drying endpoint common to all methods. The above texture indicators are important physical indicators for evaluating the quality of sheep placenta decoction pieces, which can be used to evaluate whether the drying process is standardized, in place, and uniform, thereby reflecting the intrinsic quality of sheep placenta decoction pieces. Comprehensively considering the above texture attributes, the indicators of the VFD group are mostly in a moderate state among the three drying methods, making it more suitable for daily and clinical application of sheep placenta.

### 3.3 Chemical Properties

#### 3.3.1 Hydrolyzed Amino Acid Detection

Amino acids are key nutrients in sheep placenta and serve as important indicators for evaluating protein nutritional value (Wang et al. 2023). The hydrolyzed amino acid (HAA) content of sheep placenta samples varied among different drying methods, as presented in Table 2. Seventeen amino acids were detected in samples processed by the three drying methods, comprising eight essential amino acids (EAA) and nine nonessential amino acids (NEAA), with total contents ranging from 4.03 to 104.46 mg/g. Due to the inconsistent origin of fresh sheep placenta products and different contents of the fresh products themselves, there are relatively large differences in the contents between different batches with the same drying method. The amino acid analysis of sheep placenta subjected to three drying methods revealed that its amino acid composition carries distinct flavor implications and functional potential. Glutamic acid, glycine, and aspartic acid were the three most abundant amino acids in the dried samples. Among these, glutamic acid and aspartic acid are umami‐imparting amino acids (Liu et al. 2024), which, together with sweet‐tasting amino acids such as glycine, threonine, and serine, form the main taste profile of sheep placenta. The high levels of glutamic acid and aspartic acid provide a foundation for the potential rich umami and mellow taste of sheep placenta. Bitter amino acids accounted for approximately 40% of the total composition. Overall, the proportion of sweet and umami amino acids exceeded that of bitter ones, suggesting that sheep placenta may be acceptable in taste as a food or health supplement ingredient. Moreover, different drying processes had minimal influence on this fundamental taste profile.

**TABLE 2:** Different types and contents of hydrolyzed amino acids in dried sheep placenta samples, mean (*n* = 6) ± SD.

HAA		Quantity contained (mg/g)		
		BK	HAD	VFD
EAA	Valine	27.44 ± 1.95	28.36 ± 0.97	27.65 ± 1.99
	Methionine	12.00 ± 1.14	11.96 ± 0.82	11.60 ± 1.00
	Isoleucine	16.50 ± 2.24	17.68 ± 0.92	16.15 ± 1.71
	Leucine	46.19 ± 4.02	49.29 ± 2.22	48.17 ± 4.07
	Threonine	27.77 ± 2.48	29.14 ± 1.19	28.47 ± 2.00
	Phenylalanine	24.74 ± 1.91	26.09 ± 0.93	25.45 ± 2.02
	Lysine	38.91 ± 3.57	41.99 ± 1.94	40.73 ± 2.91
	Histidine	12.83 ± 1.12	13.15 ± 0.65	13.93 ± 1.34
NEAA	Aspartic acid	54.72 ± 4.68	57.80 ± 1.68	55.95 ± 4.39
	Glutamate	97.06 ± 8.14	101.77 ± 3.08	97.61 ± 6.88
	Serine	29.76 ± 2.66	32.07 ± 1.23	31.10 ± 2.76
	Proline	47.80 ± 8.21^ab^	53.65 ± 4.55^a^	43.97 ± 6.08^b^
	Glycine	59.28 ± 7.10	60.26 ± 2.80	55.60 ± 7.91
	Alanine	44.84 ± 3.49	45.11 ± 0.90	43.02 ± 4.55
	Cysteine	4.64 ± 0.53^b^	5.25 ± 0.30^a^	5.22 ± 0.24^a^
	Arginine	37.95 ± 4.25	41.29 ± 1.45	37.62 ± 4.12
	Tyrosine	19.85 ± 2.07	20.94 ± 0.99	19.66 ± 1.32
EAA		206.37 ± 18.23	217.66 ± 9.32	212.15 ± 16.27
NEAA		395.90 ± 37.16	418.13 ± 11.13	389.75 ± 33.18
TAA		602.27 ± 54.41	635.78 ± 19.47	601.90 ± 49.26
EAA/TAA		0.343	0.342	0.352
EAA/NEAA		0.521	0.521	0.544

*Note:* Significant differences among samples were determined by analysis of variance and Duncan's multiple range test (*p* < 0.05), with different letters indicating statistically different values.

Abbreviations: BK, baking; EAA, essential amino acids; HAA, hydrolyzed amino acids; HAD, hot air drying; NEAA, nonessential amino acids; TAA, total amino acid; VFD, vacuum freeze drying.

The amino acids in sheep placenta serve as the material basis for its multiple traditional functional properties. Threonine enhances immune function and disease resistance in animals. Arginine is the exclusive precursor of endogenous nitric oxide, which is generated under the action of nitric oxide synthase and participates in regulating energy‐substrate metabolism (Wang and Ji 2013).

HAD exhibited the highest content of all 17 amino acids, with the total amino acid content also being the highest among the three groups. Both HAD and VFD had higher total EAA contents than BK, with minimal differences observed between HAD and VFD. In contrast, VFD showed the lowest total NEAA content compared to the other two methods. According to FAO/WHO standards, an ideal protein has an EAA/total amino acid (TAA) ratio close to 0.4 and an EAA/NEAA ratio greater than 0.6 (Jiang et al. 2024). Across the three drying methods, the EAA/TAA ratios were 0.342–0.352, close to the ideal 0.4, while the EAA/NEAA ratios stayed below 0.6. Although none of the groups fully met the ideal protein criteria, VFD demonstrated the closest alignment with these standards. These results indicate that the VFD method offers a significant advantage in maximally preserving the natural nutritional value of proteins. Conducted under low‐temperature and low‐oxygen conditions, this process effectively prevents the degradation of heat‐sensitive amino acids and nutrient loss caused by the Maillard reaction, thereby better maintaining the integrity and bioavailability of amino acids.

#### 3.3.2 Fast GC E‐Nose Analysis

**FIGURE 4:**
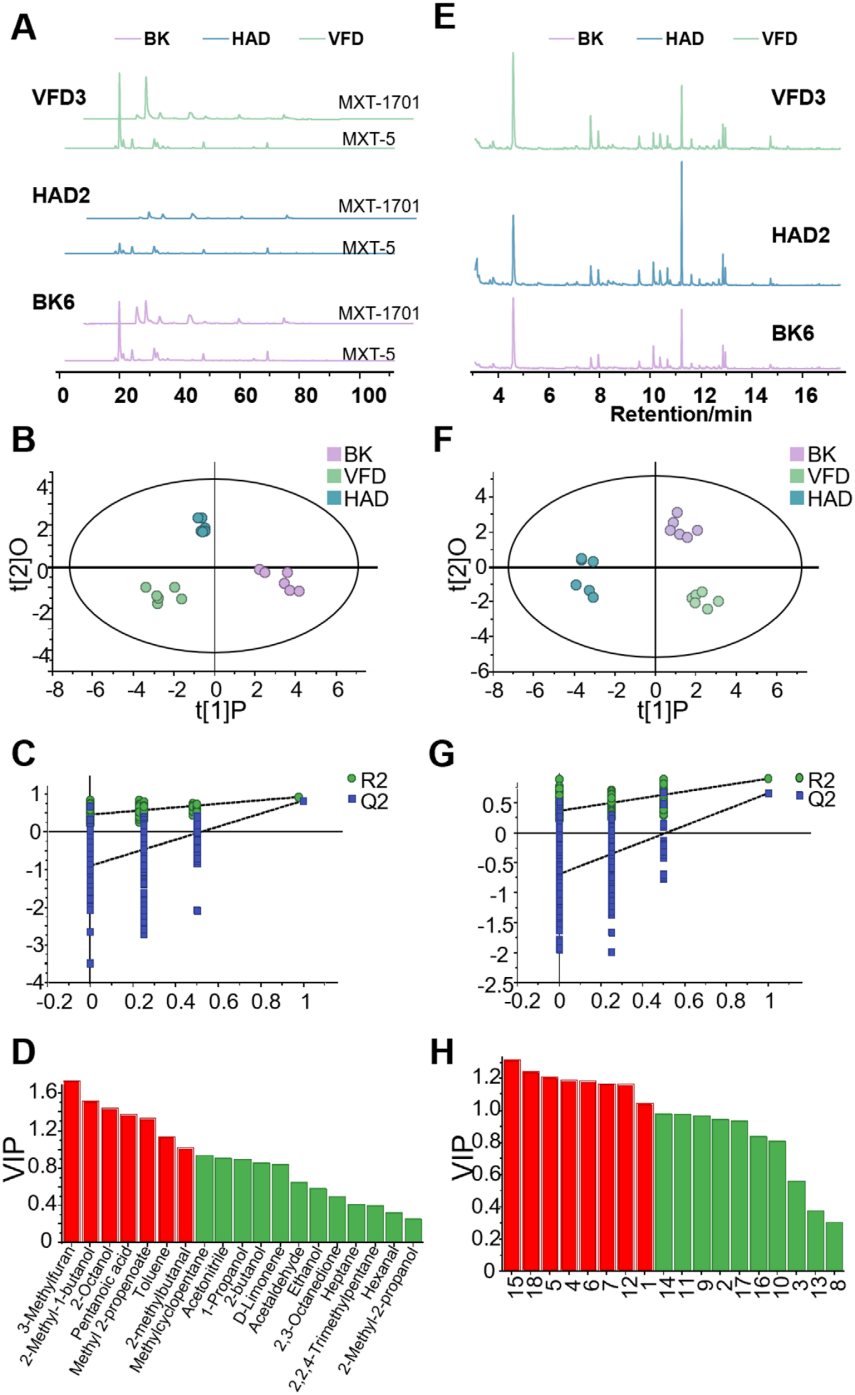
Diagrams of volatile components in different dried sheep placentas. (A) Fast GC E‐nose chromatograms of different sheep placenta powders. (B) Fast GC E‐nose OPLS‐DA. (C) Fast GC E‐nose displacement test. (D) Fast GC E‐nose VIP. (E) HS‐GC‐MS total ion chromatogram of different sheep placenta powders. (F) HS‐GC‐MS OPLS‐DA. (G) HS‐GC‐MS displacement test. (H) HS‐GC‐MS VIP. BK, baking; Fast GC E‐Nose, ultrafast gas chromatography electronic nose; HAD, hot air drying; HS‐GC‐MS, headspace gas chromatography–mass spectrometry; VFD, vacuum freeze drying.

Odor trait maps of sheep placenta with different drying methods were plotted based on the results of ultrafast gas electronic nose of each sample, including two chromatographic columns, MXT‐5 and MXT‐1701. Figure 4A shows a set of odor trait maps selected for each drying method, and the 6 sets of odor trait maps for each drying method are shown in Figure S1. The results showed that the odor Traits common components of different drying methods were relatively stable, but the odor characteristic peaks of different drying methods still had certain differences. The odor intensity of the VFD group was significantly lower than that of the other two groups, the overall odor of the BK group was higher than that of the other two groups, and the odor of individual components of the HAD group was the highest among the three groups. Qualitative analysis was performed by referring to the ArochemBase odor compound database through the relative index of the peaks of each odor characteristic component. The results are shown in Table 3. A total of 19 unique odor components were identified across the three drying methods: 18, 16, and 16 components were detected in BK, HAD, and VFD samples, respectively, with 14 components shared among all groups.

**TABLE 3:** Possible compounds and sensory description information in different dry sheep placenta samples.

NO.	Formula	MXT‐5 RI	MXT‐1701 RI	Possible compounds	Odor information	BK	HAD	VFD
1	C_2_H_4_O	439	508	Acetaldehyde	Aldehyde; Atmospheric; Fresh; Fruit; Pleasant; Pungent	+	+	+
2	C_2_H_6_O	465	561	Ethanol	Alcohol; ethanol; spicy; strong; sweet; weak	+	+	+
3	C_4_H_10_O	491	637	2‐Methyl‐2‐propanol	camphor	+	+	+
4	C_3_H_8_O	520	696	1‐Propanol	Alcohol; Ethanol; Fermented, Brewed; Fruit; Miscellaneous Alcohols; Musty; Plastic; Pungent	+	+	+
5	C_2_H_3_N	553	638	Acetonitrile	Aromatic, aromatic flavor; sweet	+	+	+
6	C_4_H_10_O	600	698	2‐Butanol	Pleasant; Intense; Sweet; Wine	+	+	+
7	C_4_H_6_O_2_	613	680	Methyl 2‐propenoate	spicy	+	−	−
8	C_5_H_6_O	626	882	3‐Methylfuran	/	+	−	+
9	C_6_H_12_	653	637	Methylcyclopentane	diesel	+	+	+
10	C_5_H_10_O	663	744	2‐Methylbutanal	Almonds; apples; burnt; burning (strong); choked; cocoa; coffee; fermented, brewed; fruits; freshly cut grass; iodoform; malt; musty; nutty; powerful; fragrant cloying odor; sour taste	+	+	+
11	C_8_H_18_	682	697	2,2,4‐Trimethylpentane	diesel	+	+	+
12	C_7_H_16_	700	698	Heptane	Alkanes; Fruit; Gasoline; Sweet	+	+	+
13	C_5_H_12_O	742	852	2‐Methyl‐1‐butanol	Alcohol; Fragrant oils, spice scents; Banana; Butter; Heteroalcohol; Iodoform; Malt; Oil; Onion (cooked); Sweet; Wine; Wine	−	−	+
14	C_7_H_8_	771	828	Toluene	Caramel; Atmosphere; Fruit; Paint; Pungent; Rubber; Solvent; Sweet; Odorless Synthetic Acid	+	+	+
15	C_6_H_12_O	802	894	Hexanal	Oak; aldehydic; oily; fishy; fresh; fruit; grassy; freshly mown; herbaceous; lush; sharp; strong; sweet; butter; wine	+	+	+
16	C_5_H_10_O_2_	924	1098	Pentanoic acid	Acidic; Beefy; Cheese; Penetrating; Pungent; Rotten; Putrid; Sour; Sweet	+	+	−
17	C_8_H_14_O_2_	982	1084	2,3‐Octanedione	Aldehyde‐based; dill; earthy; greasy; mushroom	+	+	+
18	C_8_H_18_O	995	1119	2‐Octanol	Aromatic, aromatic; coconut; earthy; greasy; fresh; fresh‐cut grassy; mushroom; oily; putrid; spicy; walnut; woodsy	+	+	−
19	C_10_H_16_	1044	1070	D‐Limonene	Tangerine; Fruit; Mint flavored; Orange; Peel flavored	+	+	+

*Note:* “+” indicates that the ingredient was detected, and “−” indicates that the ingredient was not detected.

Abbreviations: BK, baking; HAD, hot air drying; VFD, vacuum freeze drying.

The odor compositions of the three dried sheep placenta samples primarily included alcohols, hydrocarbons, aldehydes, ketones, furans, and lipids. These compounds likely form during drying through the Maillard reaction, which turns early substances into aldehydes, ketones, alcohols, furans, and pyridines (Zhang, Chen, Lan, et al. 2024). Aldehydes are key components that form the characteristic flavor of sheep placenta and important contributors to meat flavor profiles (Górska‐Horczyczak et al. 2017). Among these compounds, hexanal and 2‐methylbutanal were detected in all three drying groups. Hexanal, which typically exhibits grassy, oily, and fishy odors, is a hallmark product of lipid oxidation. In contrast, 2‐methylbutanal has strong malt, cocoa, nutty, and fruity aromas. It is associated with the Strecker degradation of amino acids and serves as a critical precursor for the formation of meaty and roasted notes (Smit et al. 2009; Wang, Lu, et al. 2025). Pentanoic acid was identified in the HAD and BK groups. Its intense cheesy, putrid, and rancid odors may result from enhanced microbial activity or accelerated fat hydrolysis during hot‐air drying, potentially exerting a negative impact on the harmony of the overall odor profile. Ethanol and D‐limonene were commonly detected in all samples. As a solvent, ethanol may facilitate the co‐volatilization of other flavor compounds. In contrast, D‐limonene contributes a fresh citrus‐like fruity aroma, which plays a positive role in masking some undesirable odors.

To further characterize the odor differentiation among different dried sheep placenta samples, an OPLS‐DA model was constructed using the peak areas of the 19 identified odor components (Figure 4B). The model exhibited good fit statistics: the R^2^X was 0.905, the R^2^Y was 0.951, and the Q^2^ was 0.845. According to statistical criteria, R^2^Y and Q^2^ values exceeding 0.5 indicate acceptable model fitness for discriminative purposes. A permutation test repeated 200 times (Figure 4C), where the intercept of the Q^2^ regression line with the vertical axis was below zero, demonstrating no overfitting and reliable predictive power. These results validate the OPLS‐DA model for discriminative analysis of odor profiles among different drying methods, further confirming significant differences in volatile compositions of dried sheep placenta samples.

To identify key odor components differentiating sheep placenta samples under different drying methods and minimize intragroup variability, an OPLS‐DA model was applied to the odor profile data (Figure 4D). Seven components with VIP values > 1 were identified: Methyl 2‐propenoate, 3‐Methylfuran, 2‐Methyl‐1‐butanol, 2‐methylbutanal, Toluene, Pentanoic acid, and 2‐Octanol. Further screening via one‐way ANOVA (*p* < 0.05) revealed six discriminant odor components: methyl 2‐propenoate, toluene, pentanoic acid, 2‐methylbutanal, 2‐methyl‐1‐butanol, and 2‐octanol. Four of the detected volatile components were characterized by a pungent odor. Among these, methyl 2‐propenoate was unique to the BK group. Its pungent odor may originate from initial endogenous enzyme or microbial activity in the raw material, and this compound is prone to decomposition or volatilization during processing. In contrast, 2‐methyl‐1‐butanol was detected exclusively in VFD samples. This indicates that the distinctive low‐temperature and oxygen‐limited conditions of VFD effectively preserve such ester compounds, which contribute a pleasant wine‐like aroma and represent a key flavor advantage of the process (Mendes‐Ferreira et al. 2019). Meanwhile, pentanoic acid, associated with rancid and cheesy odors, and 2‐octanol, with mushroom and earthy notes, were either absent or present at low levels in the VFD group. These results demonstrate that the VFD process specifically retains desirable aroma compounds like 2‐methyl‐1‐butanol while strongly suppressing off‐flavor components such as pentanoic acid and 2‐octanol. 2‐octanol was identified in both the BK and HAD groups, and its mushroom‐like and fatty odor may be linked to microbial metabolism during processing. 2‐methylbutanal was present in all three drying techniques. Pentanoic acid and 2‐octanol were commonly found in the HAD group, indicating that HAD not only enhances roasted notes but also carries a higher risk of introducing off‐odors. Overall, compared to the other two methods, the VFD samples contained fewer undesirable odor compounds and showed lower instrumental response values for such components, resulting in better flavor‐masking performance.

#### 3.3.3 HS‐GC‐MS Analysis

A total of 18 volatile substances were screened and identified from different dried sheep placentas by HS‐GC‐MS detection and comparison with the NIST 20.0 L library. Among them, 16 components were common. Figure 4E shows the total ion chromatograms of one group for each drying method, and all total ion chromatograms for each drying method are shown in Figure S1C.

The volatile differential compositions in different dried samples of sheep placenta are shown in Table 4, containing six aldehydes, five hydrocarbons, three ketones, two heterocyclics, one acid, and one alcohol volatile component. Literature reports indicate that the fishy odor in animal‐derived drugs primarily originates from volatile components such as aldehydes, amines, sulfur ethers, and heterocyclic compounds (Ye et al. 2024). The volatile components of different dried sheep placenta products partially overlapped with the volatile components emitting fishy odor in animal drugs, mainly aldehydes and hydrocarbons, and amines and sulfur ethers in sheep placenta samples were not detected by the gas‐phase electronic nose and headspace plasma, which indicated that the drying treatment could improve the undesirable odor of sheep placenta appropriately.

**TABLE 4:** Volatile components of sheep placenta using different drying methods, mean (*n* = 6) ± SD.

NO.	R.T./min	CAS	Compounds	BK	VFD	HAD
1	3.17	000079‐09‐4	Propanoic acid	—	—	5.25 ± 2.94
2	3.69	000108‐88‐3	Toluene	0.36 ± 0.55^b^	1.04 ± 0.28^a^	1.04 ± 0.20^a^
3	3.81	000071‐41‐0	1‐Pentanol	2.79 ± 0.24	2.65 ± 0.32	2.93 ± 0.31
4	4.60	000066‐25‐1	Hexanal	33.7 ± 2.27^a^	29.42 ± 3.45^b^	22.09 ± 1.09^c^
5	7.65	000110‐43‐0	2‐Heptanone	4.91 ± 0.57^b^	7.59 ± 1.31^a^	4.94 ± 0.18^b^
6	7.95	000111‐71‐7	Heptanal	4.79 ± 0.55^a^	4.32 ± 0.24^a^	3.44 ± 0.43^b^
7	8.28	000123‐32‐0	Pyrazine, 2,5‐dimethyl—	—	1.23 ± 0.78	—
8	9.55	000122‐78‐1	Benzaldehyde	3.24 ± 0.26	3.38 ± 1.68	3.78 ± 0.24
9	10.38	003777‐69‐3	Furan, 2‐pentyl—	4.63 ± 0.55^a^	4.92 ± 0.44^a^	3.74 ± 0.35^b^
10	10.68	000124‐13‐0	Octanal	3.13 ± 0.16^a^	3.01 ± 0.47^a^	2.58 ± 0.33^b^
11	11.15	000099‐87‐6	p‐Cymene	0.12 ± 0.18^b^	0.06 ± 0.15^b^	0.43 ± 0.07^a^
12	11.24	005989‐27‐5	D‐Limonene	13.66 ± 1.43^b^	10.36 ± 2.14^c^	17.73 ± 2.53^a^
13	11.62	000122‐78‐1	Benzeneacetaldehyde	2.28 ± 0.47	2.70 ± 0.87	2.37 ± 0.19
14	11.95	000099‐85‐4	.gamma.‐Terpinene	0.98 ± 0.31^a^	0.53 ± 0.27^b^	1.12 ± 0.26^a^
15	12.71	000821‐55‐6	2‐Nonanone	0.93 ± 0.16^b^	1.51 ± 0.18^a^	1.13 ± 0.22^b^
16	12.87	013466‐78‐9	3‐Carene	5.15 ± 1.48^ab^	3.98 ± 0.7^b^	6.04 ± 0.74^a^
17	12.96	000124‐19‐6	Nonanal	4.61 ± 0.39^a^	4.42 ± 0.69^a^	3.47 ± 0.3^b^
18	14.73	000693‐54‐9	2‐Decanone	1.79 ± 0.17^b^	3.09 ± 0.66^a^	1.52 ± 0.21^b^

*Note:* Significant differences among samples were determined by analysis of variance and Duncan's multiple range test (*p* < 0.05), with different letters indicating statistically different values. “−” indicates that the ingredient was not detected.

Abbreviations: BK, baking; HAD, hot air drying; VFD, vacuum freeze drying.

Key differential odor components in dried sheep placenta powders were screened via headspace mass spectrometry, followed by OPLS‐DA with 200 permutation tests. Volatile compounds were filtered using criteria of *p* < 0.05 and VIP > 1. As shown in Figure 4F, the model fit was strong: R^2^X was 0.829, R^2^Y was 0.929, and Q^2^ was 0.776. Model stability and validity were confirmed via permutation testing (Figure 4G), where no overfitting was observed, supporting its reliability for characterizing odor differences among drying methods. These results validate the OPLS‐DA model as an effective tool for identifying key volatile markers distinguishing sheep placenta processed by different drying techniques.

Using the OPLS‐DA model, headspace analysis results of different dried sheep placenta samples were analyzed (Figure 4H). Eight volatile components with VIP values > 1 were initially identified: propanoic acid, hexanal, 2‐heptanone, heptanal, pyrazine, 2,5‐dimethyl‐, D‐limonene, 2‐nonanone, and 2‐decanone. Following further screening with a significance criterion of *p* < 0.05, six differential volatile components were retained: hexanal, 2‐heptanone, pyrazine, 2,5‐dimethyl‐, D‐limonene, 2‐nonanone, and 2‐decanone. These components can be considered key volatile flavor markers distinguishing sheep placenta processed by different drying methods. Three of the above six volatile flavor components are ketones, and the increase in ketones is caused by the degradation of unsaturated fatty acids (Ni et al. 2023). As can be seen from Table 4, three ketones were detected in all three drying methods: 2‐heptanone is a compound with a fruity and soapy aroma, 2‐nonanone has a cheesy and musty smell, and 2‐decanone has a fruity smell, and all three components made a good contribution to the improvement of the flavor of the dried sheep placenta. Hexanal and D‐limonene were the most abundant volatile components in all three types of dried sheep placenta samples. Hexanal, characterized by a greasy and fishy odor, is considered the key compound contributing to the fatty taste and potential off‐odor of sheep placenta. Although hexanal remained the predominant volatile after drying, its relative content decreased in all processed samples. The lowest hexanal content was observed in the HAD group, followed by the VFD group. This reduction may be explained by the further oxidation or conversion of hexanal into other derivatives during the HAD process. D‐Limonene, which has an “orange peel flavor,” can also greatly improve the flavor of sheep placenta after drying. Pyrazine, 2,5‐dimethyl‐, which is unique to the VFD group, has a “roasted nutty flavor,” which is one of the reasons why the odor of VFD sheep placenta is different from that of the other two drying methods. This is also the key differentiating factor between the odor of VFD sheep placenta and the flavor of the other two drying methods.

The level of volatile component content is not the only indicator for evaluating the odor trait of a sample. Aroma components with higher relative odor activity value (ROAV) will also be taken as characteristic aroma Substances (Zhang, Chen, Lan, et al. 2024). The concentration and threshold of a single component together determine its contribution to the overall aroma of the sample. An ROAV value of 100 was set as the benchmark for the most influential odor contributors, and the ROAVs of other volatile components were calculated using the following formula (Zhang, Sun, et al. 2025):

(7) $$\begin{array}{c} \text{ROAV}=100\times \frac{C}{C_{\max}}\times \frac{T_{\max}}{T} \end{array}$$

Due to its high concentration and low sensory threshold, hexanal was identified as the major odor contributor and set as the reference with ROAV = 100. The ROAV values of the remaining components were then calculated using the equation above.

Table 5 shows that 6, 7, and 9 volatile flavor compounds with ROAVs ≥ 1 were present in the BK, VFD, and HAD samples, respectively, suggesting that these constituents made an important contribution to the odor of sheep placenta (Huang et al. 2018). Among the constituents with ROAVs ≥ 1, the VFD samples had more 2‐Heptanone with fruity and soapy flavors than the other two groups, which contributed to the improvement of the odor of VFD sheep placenta. The HAD group had more benzaldehyde, propanoic acid, and p‐cymene than the other two groups, with almond, caramel, spicy, cheese, and aromatic flavors. Propionic acid and p‐Cymene were unique to drying and had high aroma activity values, but both components had a pungent odor, which resulted in a higher pungent odor of the HAD sheep placenta than the other two drying components. Among the six different components mentioned above, hexanal, D‐limonene, and 2‐heptanone had high aroma activity values, and these three components were also the most critical components that caused the difference in aroma between different drying methods of sheep placenta.

**TABLE 5:** Differences in aroma components ROAV of different dried sheep placenta.

Species	Compounds	OT (μg/kg)	Fragrance description	ROAV		
				BK	VFD	HAD
Alcohols	1‐Pentanol	0.1502	Balm	16.54	17.99	26.49
Aldehydes	Hexanal	0.3	Fishy, buttery odor	100	100	100
	Heptanal	0.2	Fruity at low concentrations, rancid, fishy or fatty at high concentrations	21.32	22.03	23.36
	Benzaldehyde	4	Almond flavor, caramel flavor	0.72	0.86	1.28
	Octanal	0.4	Rancid, soapy odor	6.97	7.67	8.76
	Benzeneacetaldehyde	4	sweet	0.51	0.69	0.80
	Nonanal	0.3	Grassy at low concentrations, rancid, fishy or fatty at high concentrations	13.68	15.02	15.71
Acids	Propanoic acid	1	Spicy, cheesy flavor	—	—	7.13
Hydrocarbons	Toluene	98	Pungent and pungent flavor	< 0.01	0.01	0.01
	p‐Cymene	0.12	Aromatic, Aromatic; Fragrant Oil's, Spice Scent; Orange; Fresh; Fruit; Fuel Flavor; Gasoline; Herbal; Lemon; Mild; Pleasant; Solvent; Spicy; Sweet; Weak; Woody	—	—	4.87
	D‐Limonene	10	Delightful lemon flavor	1.22	1.06	2.41
	.gamma.‐Terpinene	1000	Citrus and woody notes	< 0.01	< 0.01	< 0.01
	3‐Carene	8.6	Turpentine odor with slight citrus notes	0.53	0.47	0.95
Ketones	2‐Heptanone	6.8	Fruity, Soap Fragrance	0.64	1.14	0.99
	2‐Decanone	110	fruity	0.01	0.03	0.02
	2‐Nonanone	40	Smells like cheese, mold	0.02	0.04	0.04
Heterocyclic	Pyrazine, 2,5‐dimethyl—	1800	Roasted Nut Flavor	—	< 0.01	—
	Furan, 2‐pentyl—	6	stinks	0.69	0.84	0.85

Abbreviations: BK, baking; HAD, hot air drying; ROAV, relative odor activity value; VFD, vacuum freeze drying.

Although 2‐nonanone and 2‐decanone exhibited low ROAV values, their contents were significantly higher in the VFD group compared to the other two methods. This indicates that the VFD conditions may specifically favor the formation or retention of these lipid oxidation derivatives. Meanwhile, both the content and ROAV value of 2‐heptanone were highest in the VFD group. Together, these ketones contribute to the distinct ketonic aroma profile of the VFD samples. Pyrazine, 2,5‐dimethyl, an important Maillard reaction product associated with a roasted nutty aroma (Zhang et al. 2022), was detected only in VFD samples. This suggests that the low‐temperature and low‐pressure environment of VFD does not inhibit the Maillard reaction, but may instead promote the generation of such characteristic pyrazines, possibly due to the extended drying duration or other specific mechanisms. This provides key evidence for the roasted aroma notes in VFD‐processed products. In addition, the alkene D‐limonene has a high odor threshold, resulting in a limited contribution to the overall ROAV. Its primary role is to modify and harmonize the general odor profile.

Combined detection using a Fast GC E‐Nose and HS‐GC‐MS identified 34 volatile components, as summarized in Table 6. Both methods consistently detected toluene, D‐limonene, and hexanal. D‐Limonene and hexanal showed the highest relative contents among all volatiles and were key contributors to flavor differences. This indicates good agreement between the two techniques, though their compound coverage differs. For instance, the electronic nose responded to several alcohol compounds, whereas only one alcohol was identified by HS‐GC‐MS. Therefore, combining both methods provides a more comprehensive analysis of volatile flavor profiles in dried sheep placenta products.

**TABLE 6:** Volatile compounds and their odor characteristics.

NO.	Compounds	Odor information	BK	HAD	VFD
1	Acetaldehyde	Aldehyde; Atmospheric; Fresh; Fruit; Pleasant; Pungent	+	+	+
2	Ethanol	Alcohol; ethanol; spicy; strong; sweet; weak	+	+	+
3	2‐Methyl‐2‐propanol	Camphor	+	+	+
4	1‐Propanol	Alcohol; Ethanol; Fermented, Brewed; Fruit; Miscellaneous Alcohols; Musty; Plastic; Pungent	+	+	+
5	Acetonitrile	Aromatic, aromatic flavor; sweet	+	+	+
6	2‐Butanol	Pleasant; Intense; Sweet; Wine	+	+	+
7	Methyl 2‐propenoate	Spicy	+	−	−
8	3‐Methylfuran	/	+	−	+
9	Methylcyclopentane	Diesel	+	+	+
10	2‐Methylbutanal	Almonds; apples; burnt; burning (strong); choked; cocoa; coffee; fermented, brewed; fruits; freshly cut grass; iodoform; malt; musty; nutty; powerful; fragrant cloying odor; sour taste	+	+	+
11	2,2,4‐Trimethylpentane	Diesel	+	+	+
12	Heptane	Alkanes; Fruit; Gasoline; Sweet	+	+	+
13	2‐Methyl‐1‐butanol	Alcohol; Fragrant oils, spice scents; Banana; Butter; Heteroalcohol; Iodoform; Malt; Oil; Onion (cooked); Sweet; Wine; Wine	−	−	+
14	Toluene	Caramel; Atmosphere; Fruit; Paint; Pungent; Rubber; Solvent; Sweet; Odorless Synthetic Acid	+	+	+
15	Hexanal	Oak; aldehydic; oily; fishy; fresh; fruit; grassy; freshly mown; herbaceous; lush; sharp; strong; sweet; butter; wine	+	+	+
16	Pentanoic acid	Acidic; Beefy; Cheese; Penetrating; Pungent; Rotten; Putrid; Sour; Sweet	+	+	−
17	2,3‐Octanedione	Aldehyde‐based; dill; earthy; greasy; mushroom	+	+	+
18	2‐Octanol	Aromatic, aromatic; coconut; earthy; greasy; fresh; fresh‐cut grassy; mushroom; oily; putrid; spicy; walnut; woodsy	+	+	−
19	D‐Limonene	Tangerine; Fruit; Mint flavored; Orange; Peel flavored	+	+	+
20	Propanoic acid	Spicy, cheesy flavor	−	+	−
21	1‐Pentanol	Balm	+	+	+
22	2‐Heptanone	Fruity, Soap Fragrance	+	+	+
23	Heptanal	Fruity at low concentrations, rancid, fishy or fatty at high concentrations	+	+	+
24	Pyrazine, 2,5‐dimethyl—	Roasted Nut Flavor	−	+	−
25	Benzaldehyde	Almond flavor, caramel flavor	+	+	+
26	Furan, 2‐pentyl—	Stinks	+	+	+
27	Octanal	Rancid, soapy odor	+	+	+
28	p‐Cymene	Aromatic, Aromatic; Fragrant Oil's, Spice Scent; Orange; Fresh; Fruit; Fuel Flavor; Gasoline; Herbal; Lemon; Mild; Pleasant; Solvent; Spicy; Sweet; Weak; Woody	+	+	+
29	Benzeneacetaldehyde	Sweet	+	+	+
30	.gamma.‐Terpinene	Citrus and woody notes	+	+	+
31	2‐Nonanone	Smells like cheese, mold	+	+	+
32	3‐Carene	Turpentine odor with slight citrus notes	+	+	+
33	Nonanal	Grassy at low concentrations, rancid, fishy or fatty at high concentrations	+	+	+
34	2‐Decanone	Fruity	+	+	+

*Note:* “+” indicates that the ingredient was detected, and “−” indicates that the ingredient was not detected.

Abbreviations: BK, baking; HAD, hot air drying; VFD, vacuum freeze drying.

### 3.4 Comprehensive Analysis

#### 3.4.1 Correlation Analysis

**FIGURE 5:**
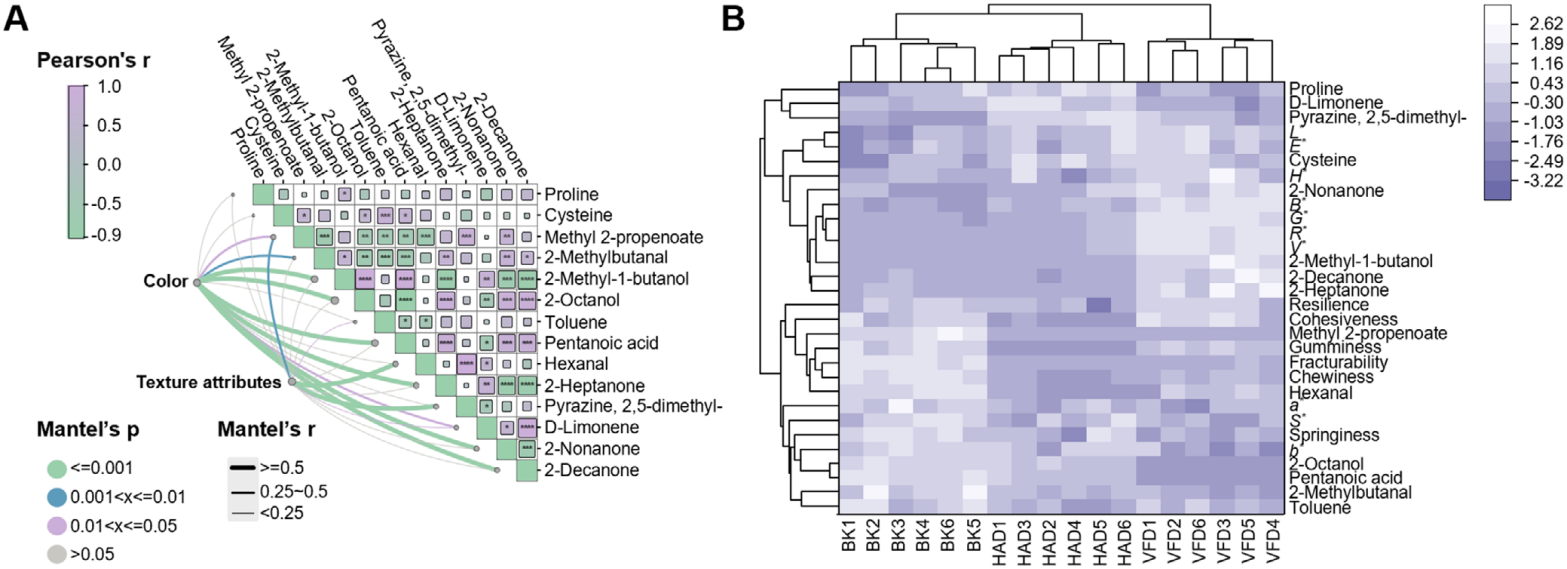
Correlation analysis. (A) Analysis of color, texture attributes, and compound correlation. (B) Cluster analysis of different dry sheep placenta colors, texture attributes, and compounds. a*, red‐green chromaticity; B*, blue value; b*, yellow‐blue chromaticity; BK, baking; E*, the total color difference; G*, green value; H*, hue; HAD, hot air drying; L*, lightness value; R*, red value; S*, saturation; V*, value; VFD, vacuum freeze drying.

Correlation analysis of the color, texture attributes, differentially hydrolyzed amino acids, and differentially flavored compounds of sheep placenta (Figure 5A) showed that there were mostly significant correlations between flavor components, such as 2‐heptanone shows a significant positive correlation with 2‐nonanone and 2‐decanone; all three ketones have odor‐improving effects. Propionic acid shows a significant negative correlation with 2‐nonanone and 2‐decanone, indicating that undesirable odors can affect the content of aromatic odor components. Cysteine shows a significant negative correlation with toluene, which may be related to toluene undergoing oxidative metabolism after the sheep placenta is dried, leading to a decrease in cysteine content. Color and texture attributes also exhibit significant correlations with volatile flavor compounds. Thicker lines indicate a stronger correlation between the two. Color shows a strong association with 2‐Methyl‐1‐butanol, 2‐octanol, pentanoic acid, 2‐nonanone, and others, suggesting that color is significantly correlated with specific volatile components. Similarly, texture attributes show a strong association with Pyrazine, 2,5‐dimethyl‐ and hexanal. However, the correlations of color and texture attributes with cysteine and proline content were significantly low and statistically insignificant, indicating that the aforementioned amino acids are independent of color and texture attributes. In summary, Figure 5A clearly presents the multidimensional association of color‐texture with composition, providing a basis for analyzing the synergistic mechanisms of substances and constructing quality prediction models.

The color, texture attributes, and composition data of different dried sheep placenta samples were subjected to cluster analysis after z‐score standardization. The different dried sheep placenta samples were clustered separately based on color, texture attributes, and composition. Figures are shown in Figure S2. Only the composition could effectively cluster the three drying methods into one category. For both color and texture attributes, only the freeze‐dried group could be well clustered into one category, while the other two drying methods could not be clustered into one category. Finally, a total cluster analysis was performed on the color, texture attributes, and composition of the three drying methods. Figures are shown in Figure 5B. The oven‐dried group, the freeze‐dried group, and the roasted group could all be well clustered into one category. Lighter colors indicated positive correlations, whereas darker shades represented negative associations. The VFD group was mostly positively correlated with color, 2‐Methyl‐1‐butanol, and 2‐nonanone, indicating that VFD better preserved the color of sheep placenta samples, which may be related to the reduction of the Maillard and oxidative reactions by the low‐temperature vacuum. The BK group was negatively correlated with color, amino acid composition and aromatic components. It can be seen from this that the improvement in color, amino acid retention and odor of sheep placenta extract after baking has little correlation with the baking of sheep placenta. The HAD group showed mostly negative correlations with most of the indicators, which may be related to the occurrence of thermal decomposition or the Maillard reaction due to high temperature.

#### 3.4.2 Comprehensive Evaluation

We applied the TOPSIS method. The z score values for color, texture, amino acids and flavor markers from each dried sheep placenta slice served as the indicators. For the comprehensive evaluation model of sheep placenta decoction pieces using different drying methods, the a* and b* values increase after oxidation and Maillard reactions, and lower saturation results in more visually appealing products. Therefore, these three indicators are treated as negative indicators, while all other color indicators are positive indicators. A smaller fracturability value indicates a crispier sample; gumminess and chewiness show the same trend as fracturability; and a smaller springiness value means easier chewing. Consequently, fracturability, gumminess, chewiness, and springiness are classified as negative indicators, whereas cohesiveness and resilience are positive indicators. For flavor components, methyl 2‐propenoate, 2‐methylbutanal, toluene, pentanoic acid, and hexanal, which contribute to pungent and fishy odors, are considered negative indicators. In contrast, 2‐methyl‐1‐butanol, 2‐heptanone, 2,5‐dimethyl‐pyrazine, D‐Limonene, 2‐nonanone, and 2‐decanone, which produce aromatic odors, are treated as positive indicators. The entropy weight method was employed to determine the weight of each indicator. The model then ranked the slices from the different drying methods. According to the following formula (Gülmez 2025):

(8) $$\begin{array}{c} D_{i}^{+}=\sqrt{\sum_{i=1}^{j}{\left(R_{\mathrm{ij}}-V^{+}\right)}^{2}} \end{array}$$

(9) $$\begin{array}{c} D_{i}^{-}=\sqrt{\sum_{i=1}^{j}{\left(R_{\mathrm{ij}}-V^{-}\right)}^{2}} \end{array}$$

(10) $$\begin{array}{c} C_{i}=\frac{D_{i}^{-}}{D_{i}^{-}+D_{i}^{+}} \end{array}$$

Calculate the distances of 18 groups of drying methods from the distances of alternatives to positive ideal solution (*D* ^+^_i_), the distances of alternatives to negative ideal solution (*D* ^−^_i_), and calculation of the closeness coefficient (*Ci*). When *Ci* is between 0 and 1, the larger the value, the better the quality of the sample. The results are shown in Table 7 The TOPSIS evaluation results are VFD group > HAD group > BK group. The Ci degrees of the VFD group were all greater than 0.6, indicating that the quality of the VFD group of sheep placenta slices is far better than that of the HAD and BK sheep placenta slices.

**TABLE 7:** TOPSIS evaluation models of different dried sheep placentas.

Group	*D* ^+^_*i*_	*D* ^−^_*i*_	*Ci*	Sort
BK1	0.81	0.377	0.31	13
BK2	0.84	0.31	0.27	14
BK3	0.85	0.26	0.23	16
BK4	0.84	0.28	0.25	15
BK5	0.88	0.26	0.23	17
BK6	0.87	0.25	0.22	18
VFD1	0.38	0.75	0.66	4
VFD2	0.37	0.74	0.66	3
VFD3	0.29	0.84	0.74	1
VFD4	0.41	0.75	0.65	5
VFD5	0.38	0.76	0.67	2
VFD6	0.44	0.70	0.61	6
HAD1	0.73	0.46	0.39	12
HAD2	0.72	0.48	0.40	10
HAD3	0.73	0.47	0.39	11
HAD4	0.71	0.51	0.42	9
HAD5	0.68	0.51	0.43	8
HAD6	0.67	0.52	0.44	7

Abbreviations: BK, baking; *Ci*, calculation of the closeness coefficient; *D* ^−^_i_, the distances of alternatives to negative ideal solution; *D* ^+^_i_, the distances of alternatives to positive ideal solution; HAD, hot air drying; VFD, vacuum freeze drying.

## 4 Discussion

This study systematically evaluated the effects of vacuum freeze drying, hot air drying, and baking on the overall quality of sheep placenta decoction pieces. It revealed intrinsic relationships between drying methods, appearance characteristics, physicochemical properties, and flavor profiles. By integrating intelligent sensing technologies such as machine vision, Fast GC E‐Nose, and texture analysis with modern analytical techniques including SEM, LF‐NMR, HS‐GC‐MS, and amino acid analysis, a multidimensional quality evaluation system covering color, aroma, texture, and composition was established. This system combines traditional experience with modern scientific methods to characterize sheep placenta as a functional food. Using Mantel test, Pearson correlation analysis, and the TOPSIS model, a comprehensive assessment was conducted to objectively evaluate the advantages and limitations of different drying methods. The findings provide a scientific basis for the streamlined processing of sheep placenta.

Different drying methods significantly influenced the quality of sheep placenta samples. VFD samples showed the closest appearance to fresh material, the most desirable texture, and the best microstructure preservation. The low‐temperature and low‐oxygen conditions of this method help maintain structural integrity while effectively inhibiting thermal degradation and lipid oxidation (Tong et al. 2025; Yang, Jia, et al. 2025). As a result, VFD samples contained lower relative levels of off‐odor compounds such as hexanal and pentanoic acid. These conditions also promoted a mild and selective Maillard reaction, leading to the unique presence of pyrazine, 2,5‐dimethyl‐ only in the VFD group. Additionally, VFD better preserved volatile alcohols like 2‐methyl‐1‐butanol. Therefore, the VFD process produces a fresh flavor profile characterized by roasted nutty and fruity notes, with significantly reduced fishy and unpleasant odors. Although VFD involves higher equipment and energy costs (Ma et al. 2018), it is suitable for developing high‐end natural products with a pure flavor, which can enhance product quality and market competitiveness. HAD uses high‐speed airflow and convective heat transfer to remove moisture from samples (Wang, Chen, et al. 2025). While controllable and easy to use, this method can cause thermal degradation of heat‐sensitive compounds and promote Maillard reactions. This led to darker color and uniform disruption of microstructure in HAD samples. Maillard reactions also contribute to the loss of active components, affecting both nutritional and sensory qualities (Mu et al. 2025; Park et al. 2019). For instance, the HAD group exhibited higher levels of hexanal and 2‐methylbutanal, creating a rich fatty‐roasted flavor. Higher temperatures also promoted Strecker degradation of amino acids and further oxidation of lipids, generating short‐chain fatty acids such as pentanoic acid. Additionally, nonanal showed a high ROAV value, contributing to potential off‐odors. Thus, the flavor profile of HAD products is more intense and complex, making this method suitable for products targeting traditional and robust flavors. Bk involves higher temperatures and an unstable thermal environment, which can induce radical formation, deplete antioxidants, accelerate lipid oxidation, and negatively affect product quality (Shen et al. 2022). BK samples showed uneven coloring, significant surface damage under microscopy, and a tendency to form complex and sometimes unbalanced volatile compound combinations. This increases the difficulty of achieving consistent flavor control. Therefore, baking is more suitable for traditional preparation methods that target specific flavor profiles, rather than serving as a core process for modern standardized production.

Sheep placenta is an animal‐derived functional food containing predominantly heat‐sensitive protein components. Although the HAD samples showed the highest total amino acid content, lipid oxidation and enzymatic reactions during processing likely caused the denaturation or inactivation of thermolabile amino acids, ultimately reducing sample quality (Dai et al. 2025). In contrast, the essential to total amino acid ratio of the VFD group was closest to the ideal protein benchmark of 0.4. This is attributed to the low‐temperature conditions of VFD, which minimize Maillard‐related consumption of essential amino acids and reduce heat‐induced protein denaturation (Mu et al. 2025). Owing to its mild processing, VFD also best preserves the integrity of heat‐sensitive functional amino acids such as glutamic acid, glycine, alanine, and proline. In comparison, the high‐temperature treatments of hot air drying and baking increase the risk of degradation or structural alteration of these functional components (Yadav et al. 2025). Natural biological variation in fresh sheep placenta may cause batch‐to‐batch differences in composition. Nevertheless, samples from the same origin exhibited consistent overall trends across the different drying methods. Future research using a larger sample set from diverse origins will help further validate and generalize these findings.

## 5 Conclusion

This study systematically evaluated the effects of vacuum freeze drying, hot air drying, and baking on the quality of sheep placenta extracts. Results showed that vacuum freeze drying outperformed the other methods in preserving heat‐sensitive components, reducing off‐flavors, enhancing pleasant aromas, and maintaining structural integrity. Collectively, these advantages improve the sensory, nutritional, and commercial quality of the product, providing a robust scientific basis for developing sheep placenta‐based functional foods marketed with the selling point of “mild flavor and complete composition” and facilitating their application in high‐end functional foods. Compared with traditional drying methods, VFD technology has higher energy consumption and costs. Future research can optimize VFD curves or combine VFD with energy‐efficient technologies such as microwaves and heat pumps to reduce energy consumption and costs while ensuring product quality. This approach will enable more sustainable industrial production and promote green and low‐carbon processing, which is of great significance for achieving the “carbon neutrality” goal in food processing. In conclusion, vacuum freeze drying demonstrates strong potential for enhancing the quality of sheep placenta and similar materials. Further technological improvements are expected to expand its role in the health‐conscious food industry.

## Author Contributions

Jing Zhu: Conceptualization, formal analysis, writing – original draft, writing – review and editing, project administration, and funding acquisition. Yuqing Fan: methodology, software, writing – original draft, and writing – review and editing. Jiale Du: Methodology and investigation. Xingxing Liu: Visualization and formal analysis. Guisheng Yi: Methodology. Hongmin Yu: Software. Jingrong Fu: Resources. Jinghong Fu: Resources. Lingyun Zhong: Supervision. Ming Yang: Supervision and investigation. All authors have read and agreed to the published version of the manuscript.

## Funding

This work was supported by the Jiangxi Provincial Key R&D Program‐Industrial Chain Science and Technology Innovation Consortium Unveiled and Commanded Project (No. 20224BBG71022); Jiangxi Province “Double Thousand Talents” High‐end Talent Program for Youth in Science and Technology Innovation (No. xsg2023201117); Yichun Science and Technology Program Project (No. Yike Zi [2024] No. 17); Jiangxi University of Traditional Chinese Medicine Artisan Technique Heritage Innovation Team Project (No. CXTD22003); Jiangxi University of Traditional Chinese Medicine Graduate Innovation Special (No. XJ‐S202484); Jiangxi University of Traditional Chinese Medicine Undergraduates’ Innovative and Entrepreneurial Training Program (No. 202410412191).

## Conflicts of Interest

Authors Jingrong Fu and Jinghong Fu from Jiangxi Tianyuan Pharmaceutical Co. Ltd. only provided the sample resources for the study but did not participate in the article's writing. The remaining authors declare that no business or financial relationship that could be considered as a potential conflicts of interest existed during the conduct of this study. Any commercial or financial relationship that could be perceived as a potential conflict of interest.

## Supporting information


**Figure S1:** Chromatograms of sheep placenta odor by different drying methods. (A) Fast GC E‐Nose MXT‐5. (B) Fast GC E‐Nose MXT‐1701. (C) HS‐GC‐MS. BK, Baking, Fast GC E‐Nose, ultrafast gas chromatography electronic nose; HAD, hot air drying; HS‐GC‐MS, headspace gas chromatography‐mass spectrometry; VFD, vacuum freeze drying.
**Figure S2:** (A) Cluster analysis of different dry sheep placenta colors. (B) Cluster analysis of different dry sheep placenta texture attributes. (C) Cluster analysis of different dry sheep placenta compounds. a*, red‐green chromaticity; B*, blue value; b*, yellow‐blue chromaticity; BK, baking; E*, the total color difference; G*, green value; H*, hue; HAD, hot air drying; L*, lightness value; R*, red value; S*, saturation; V*, value; VFD, vacuum freeze drying.

## Data Availability

The data that support the findings of this study are available from the corresponding author upon reasonable request.
