# Supplementary material for: Evaluation of Odor and Physicochemical Properties in Sheep Placenta Processed With Different Drying Methods
Source: Food Sci Nutr. 2026 Mar 15;14(3):e71641. doi: 10.1002/fsn3.71641 (PMC13093413; doi:10.1002/fsn3.71641)
Supplement: Supplementary file 1 — Figure S1: Chromatograms of sheep placenta odor by different drying methods. (A) Fast GC E‐Nose MXT‐5. (B) Fast GC E‐Nose MXT‐1701. (C) HS‐GC‐MS. BK, Baking, Fast GC E‐Nose, ultrafast gas chromatography electronic nose; HAD, hot air drying; HS‐GC‐MS, headspace gas chromatography‐mass spectrometry; VFD, vacuum freeze drying. Figure S2: (A) Cluster analysis of different dry sheep placenta colors. (B) Cluster analysis of different dry sheep placenta texture attributes. (C) Cluster analysis of different dry sheep placenta compounds. a*, red‐green chromaticity; B*, blue value; b*, yellow‐blue chromaticity; BK, baking; E*, the total color difference; G*, green value; H*, hue; HAD, hot air drying; L*, lightness value; R*, red value; S*, saturation; V*, value; VFD, vacuum freeze drying. [file FSN3-14-e71641-s001.docx]

**Evaluation of Odor and Physicochemical Properties in Sheep Placenta Processed with Different Drying Methods**

Jing Zhu ^1,2*^, Yuqing Fan ^1,2^, Jiale Du ^1,2^, Xingxing Liu ^1^, Guisheng Yi ^2^, Hongmin Yu ^1,2^, Jingrong Fu ^3^, Jinghong Fu ^3^, Lingyun Zhong ^2,4^, and Ming Yang ^4^

^1^ Research Center for Traditional Chinese Medicine Resources and Ethnic Minority Medicine, Jiangxi University of Chinese Medicine, Nanchang 330004, China;

^2^ School of Pharmacy, Jiangxi University of Chinese Medicine, Nanchang 330004, China;

^3^ Jiangxi Tianyuan Pharmaceutical Co., Ltd., Yichun 331208, China;

^4^ National Key Laboratory of Classic Formula Modern Chinese Medicine Creation, Nanchang 330004, China;

* Corresponding authors. E-mail address:

Jing Zhu, 20091024@jxutcm.edu.cn.

**Supplementary Figures**

**Figure S1.** **…………………………………………………………………………………… 1**

**Figure S2.** **…………………………………………………………………………………… 2**

**Supplementary Figures**

**Figure S1.** Chromatograms of sheep placenta odor by different drying methods. (A) Fast GC E-Nose MXT-5. (B) Fast GC E-Nose MXT-1701. (C) HS-GC-MS.

*Note:* Baking (BK), Hot air drying (HAD), Vacuum freeze drying (VFD), Headspace gas chromatography-mass spectrometry (HS-GC-MS), Ultra-fast gas chromatography electronic Nose (Fast GC E-Nose).


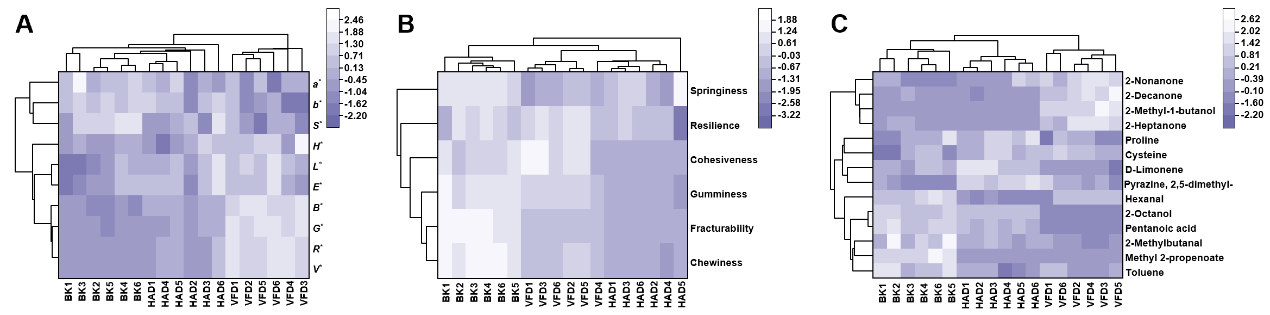


**Figure S2.** (A) Cluster analysis of different dry sheep placenta colors. (B) Cluster analysis of different dry sheep placenta texture attributes. (C) Cluster analysis of different dry sheep placenta compounds.

*Note:* Baking (BK), Hot air drying (HAD), Vacuum freeze drying (VFD), Lightness value (*L^*^*), Red-green chromaticity (*a^*^*), Yellow-blue chromaticity (*b^*^*), The total color difference (*E^*^*), Red value (*R^*^*), Green value (*G^*^*), Blue value (*B^*^*), Hue (*H^*^*), Saturation (*S^*^*), Value (*V^*^*).
